# Proximal tibial osteotomies for the medial compartment arthrosis of the knee: a historical journey

**DOI:** 10.1007/s11751-012-0131-x

**Published:** 2012-03-21

**Authors:** I. Esenkaya, K. Unay, K. Akan

**Affiliations:** Department of Orthopaedic and Traumatology, Goztepe Research and Training Hospital, Kadikoy, Istanbul, Turkey

**Keywords:** Osteotomy, Tibia, Osteoarthrosis, History

## Abstract

Several proximal tibial osteotomy techniques for the medial compartment arthrosis of the knee are described and traced in their development. These techniques are of the closed wedge, dome and open wedge types. We detail the differences in planning and surgery as well the need for different fixation devices. This historical and technical description will benefit those surgeons wishing to undertake the procedure as an alternative to joint replacement strategies.

## Introduction

Proximal tibial osteotomies are indicated for varus malalignment of the lower extremity and unicompartment medial arthrosis of the knee in young and active patients. The procedure aims to diminish load through the medial compartment. Resurgence in the use of the technique, modifications to the shape and location of the osteotomy and devices used for fixation has prompted a review of this important procedure.

## Historical perspective

Corrective osteotomies are of three principal types: a closed wedge, an open wedge or dome (barrel-vault) type. Variations exist to the location (supra- or infra-tubercle), manner of correction (whether acute or gradually achieved – hemicallotasis) and to the type of fixation device employed. Fixation devices have included plate and screws, staples, Steinmann pins, crossed Kirschner wires, Charnley compression clamps and monolateral or circular external fixators.

The proximal tibial osteotomy for degenerative arthritis of the knee was first described by Jackson [1, 2]. This was performed distal to the tibial tubercle [3] whereas Coventry [4] divided the tibia proximally and Wardle [5] at the junction of the upper and middle third of the shaft.

### Closed wedge osteotomies

**Fig. 1:**
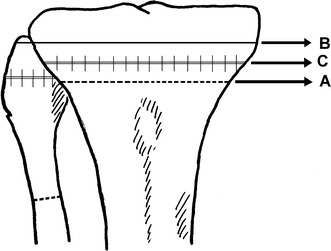
The closed wedge osteotomy lines

Göran Bauer et.al described a lateral closing wedge osteotomy performed with a transverse skin incision in the metaphyseal area proximal to the attachment site of patellar tendon. The fibula is divided separately through one of three methods: (a) an osteotomy performed 5 cm or more below the fibular neck; (b) resection of the fibular head; or (c) a separation of the tibio-fibular syndesmosis to allow the fibular head to slide proximally as the wedge is closed (Fig. 1a). They reported using a staple in their first 2 cases but omitted this later, explaining that the pressure created by the quadriceps mechanism was sufficient for stabilization. Postoperatively, a special boot named a “Unna paste boot” was utilized after 6 weeks in a cast brace [6].

Harris and Kostuik described their technique in 1970 utilizing a transverse skin incision and an osteotomy with a motorized saw 1 cm below the joint line. They stated no need to explore the common peroneal nerve or perform a fibular cut; only the styloid process is excised (Fig. 1b). The anterior height of the wedge is augmented if there was a coincident fixed flexion deformity. The wedge is closed through osteoclasis, a manoeuvre which cracked the medial cortex. Fixation is achieved with two staples with no need for supplementary external support other than a Jones bandage [7].

In 1973, Coventry described his technique utilizing either a lateral transverse or longitudinal incision. In order to displace the neurovascular structures posteriorly, the knee is flexed to at least 45°. The biceps femoris and lateral collateral ligament are separated from the fibular head with sharp dissection. The osteotomy is performed 2 cm below the joint line (Fig. 1c). The medial cortex is also fractured using the osteoclasis technique. The biceps femoris and lateral collateral ligament are then reattached to the fibula or anteriorly to the iliotibial tract. A Jones bandage and above-knee plaster slabs are applied postoperatively [4].

**Fig. 2:**
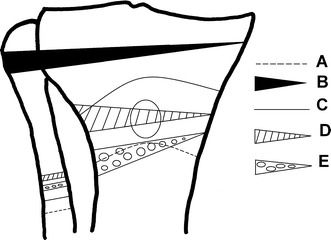
Osteotomy lines used by Jackson and Waugh over the years

Jackson and Waugh reported on six different techniques used over 20 years [8]. The first method was a curved osteotomy under the tibial tuberosity (TT) (Fig. 2a), stabilized with an above-the-knee cast for 8 weeks; the second, from 1961 onwards, involved fixation with Charnley clamps and Steinmann pins; the third, from 1964, was an osteotomy with wedge extraction from the tibial tuberosity and fixation with staples and a cast (Fig. 2b). Further changes included a curved osteotomy above the TT (Fig. 2c); then a transposition of the tibial tuberosity (Fig. 2d); and finally a wedge osteotomy below the tibial tuberosity (Fig. 2e) [8].

The authors after Jackson and Waugh described similar modifications of the osteotomy technique and choice of implants for closed wedge osteotomies. In some, the closed wedge osteotomy above the TT was fixed with a blade plate. Other osteotomies were described leaving the posterior cortex intact to extract the wedge and, then, only the lower edge of posterior cortex cut. The osteotomy line was closed with sliding of the lower edge of the proximal fragment posteriorly over the distal segment [9].

**Fig. 3:**
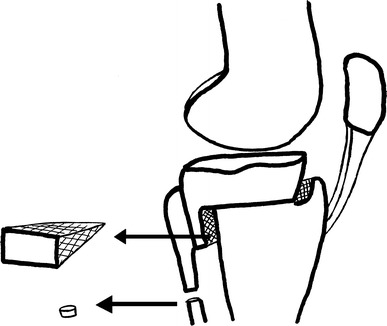
The view of interlocking wedge osteotomy reported by Ogata

In 1984, Ogata reported his technique named the “interlocking wedge osteotomy” (Fig. 3). A transverse or longitudinal incision was performed; then, through a separate incision, a fragment was excised obliquely from the proximal 1/3 of the fibula. An osteotomy parallel to the joint line was done just 5 mm proximal to the TT. A window with its base on the anterolateral cortex was opened and the upper cut taken to but not through the posterior and posteromedial cortices; the lower cut was started at the posterior cortex and directed anteriorly to but not through the anterior and anteromedial cortices. A cancellous bony wedge was then extracted and the surfaces coapted end-on-end in valgus and with 5° of internal rotation of the distal fragment with the medial cortex intact; this allows the anterior displacement of TT. In patients with advanced patellofemoral arthritis, the 5° of rotation may have been insufficient and greater advancement of the tibial tubercle in an anteromedial direction is needed. A cast was applied for 4–5 weeks postoperatively to supplement stepped staple fixation [10].

Insall et al. also performed a closed wedge osteotomy through a transverse incision with the knee in 45° of flexion. The proximal tibiofibular joint is separated and a fibular head resection or osteotomy of fibular head performed. They noted that the thinner the proximal segment of tibia, the greater the risk of avascular necrosis. They used a staple for only one patient whilst others were held in an above-the-knee cast for 2 months postoperatively [11].

Putnam et al. reported a combined technique using the Maquet and valgus tibial osteotomy. An oblique incision is used and a bilateral retinacular release with a Maquet-type tibial tubercle osteotomy (Ferguson) performed. The tibiofibular joint is separated. A closing wedge osteotomy is carried out, completed with an osteotome after pre-drilling. The extracted bone is used to elevate the TT. Internal fixation is not used and a cast applied for 6 weeks postoperatively [12]. There were also reports describing oblique wedge osteotomies based on logarithmic calculations for oblique plane corrections [13].

**Fig. 4:**
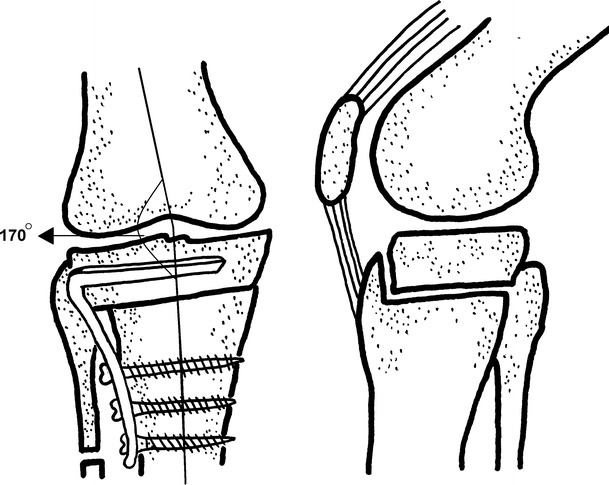
The osteotomy reported by Koshino et al. The frontal (**a**) and the lateral (**b**) view of the osteotomy

Koshino et al. [14] reported their technique in 1989. Firstly, a 3.5 cm segment is excised from the middle third of the fibula. Another anterior incision 15 cm long, convexity facing lateral, is made for the tibia. The medial and lateral retinaculum and the capsule are cut longitudinally and, in the presence of a flexion contracture, the pes anserinus elevated subperiosteally. A Kirschner wire is directed from anterolateral to posteromedial at a 30° or 15° angle. The proximal fragment is kept at least 25 mm in width with a part of the TT left as a collar. A blade plate, with 30 or 15° posterior angulation of the blade and 15° of retroversion of the body, is then applied in accordance to the size of the deformity. The distal fragment is placed at 5–10° external rotation with 2 mm medial displacement and anteriorly at a distance equal to the collar left from the TT (Fig. 4).

**Fig. 5:**
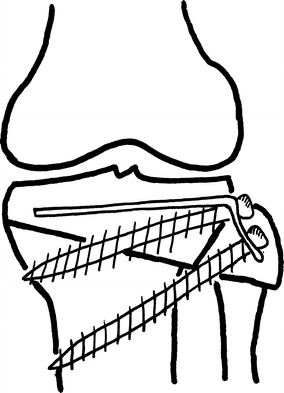
The view of modified Weber Technique reported by Miniaci et al. and the used plate-screws

Miniaci et al. [15] reported the “Modified Weber Technique.” In this technique, the incision is anterolateral. A step-cut osteotomy is performed for the fibula. A ½ tubular plate is used; the plate is bent 110° starting from the 4th hole and 20° to the other side at the 5th hole. An oblique osteotomy from lateral to medial direction is performed. A cortico-periosteal hinge is left on the medial side. A long cortical screw is placed from the 5th hole without crossing the osteotomy line. A cortical or cancellous screw serves to compress the osteotomy line from the 4th hole (Fig. 5).

Nakhostine et al. described plastic deformation of the tibial plateau as part of the technique in 1993. The iliotibial tract is divided at Gerdy’s tubercle in this procedure. The osteotomy starts 4 cm distal to lateral tibial plateau and the direction is first defined with Kirschner wires directed obliquely to the medial border of tibial plateau. The fibula is osteotomized through a separate incision at the junction of middle and upper proximal third. The osteotomy is carried out over the K wires and the medial cortex left intact. A trapezoidal wedge is removed. With an AO/ASIF L plate, the two proximal screws were driven parallel to the joint surface whereas the third is directed parallel to the osteotomy line. With the aid of the plastic deformation of the medial cortex through use of the AO/ASIF tensioning device, the osteotomy is closed and then distal screws applied to complete the fixation [16].

In their reported series in 1996, Zenger et al. [17] used an anterolateral incision to perform a closed wedge technique above TT with an oblique fibular osteotomy. They also reported drilling to weaken the medial cortex and fixed the osteotomy with two staples but, if the medial cortex was broken, a lag screw was used. In 1999, Gautier et al. [18] used a cannulated blade plate and an AO compression plate to close the osteotomy line.

The osteotomy described by Khan and Matthews in 2000 used a transverse incision. The osteotomy is performed with maximum knee flexion. The fibula is left intact because the osteotomy is located at the proximal part of the tibiofibular joint. No internal fixation is used and a long leg cast applied for 6–8 weeks [19].

The Box osteotomy described by Agarwala et al. in 2001 also used a transverse lateral incision with the knee held in approximately 90° of flexion. The peroneal nerve is not explored. The anteroinferior part of the fibular head is excised and proximal tibiofibular joint removed. A trapezoidal-shaped osteotomy is performed on the lateral tibial cortex. The upper cut is made parallel to the joint; both upper and lower cuts are made to the medial cortex. The TT is left on the distal fragment with the posterior part curved to fit the anterior part of the trapezoid. The posterior fragment is shifted to posterior and lateral to cover the osteotomy surface. Offset staples are used for fixation. The graft obtained from the wedge is then placed around the staple [20].

Several other osteotomies have also been described; examples include a “two level gap osteotomy” [21], the buttress AO plate lateral wedge osteotomy [21], the retrotubercle osteotomy [22, 23]. Plate and rigid internal fixation were applied in closed wedge osteotomies [24, 25]. Other fixation devices used in closed wedge osteotomies were: cannulated blade plates (Hippocrates), NexGen (Zimmer) osteotomy plate, Chambat plate, double plates, the “L” shaped buttress plate and cancellous/cortical screws, metal wires and the VCO Compression System [24, 26].

### Fibular osteotomies

The intact fibula causes a problem for laterally based closed wedge osteotomies in the proximal tibia. Therefore, each author has described different techniques. These techniques are diaphyseal osteotomies, resection of the fibular head, the excision of the superior tibiofibular ligaments, fibular neck osteotomy, extraction of a fibular 1–2 cm diaphyseal segment and fibular head enucleation [6]. The proximity of the common peroneal nerve requires care in proximal fibula techniques. The extensor hallucis longus motor branches can be severed in fibular osteotomies in some patients. The area of lowest risk is 40–68 mm and 160 mm distal to the fibular head [27].

### Dome (barrel-vault) osteotomy

**Fig. 6:**
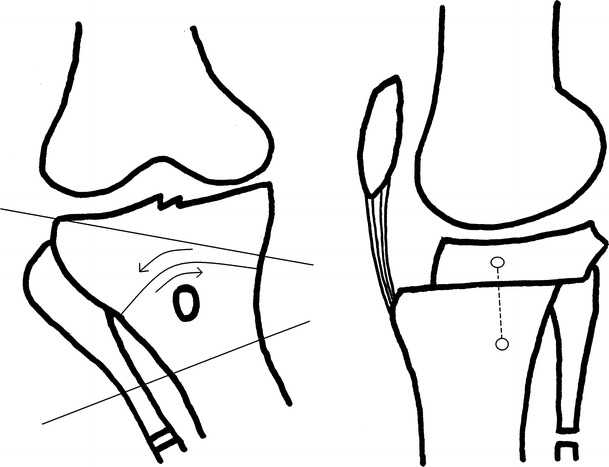
The Steinmann pins used for reduction and the view of the dome osteotomy (**a**). The lateral view of the osteotomy and Steinmann pins after reduction (**b**)

This osteotomy is known also as the barrel-vault osteotomy. It can be used with knee subluxation and deformities exceeding 15°. A tourniquet was not advised with this technique in order to reduce venous stasis, thrombophlebitis or the possible risk of pulmonary emboli. A one cm bone resection from the upper third of the fibula is performed through a posterolateral incision. A 5 cm longitudinal incision is made from TT; a perforation of the TT with a K wire is then made with the opening facing distally (concave distally). Two to four degrees are added to the existing size of deformity and a Steinmann pin driven into the tibial plateau based on this calculation. A second Steinmann pin is then driven from the distal part of the osteotomy but perpendicular to the diaphyseal axis. The proximal pin is 1–2 cm anterior to the distal one. A bony cut is made with a thin osteotome and the tibia rotated until the Steinmann pins are in line (Fig. 6). This manoeuvre shifted the distal fragment slightly to the anterior. A posterior capsulotomy is added if there was a knee flexion contracture. Compression is applied across the osteotomy with two Charnley clamps on the Steinmann pins. Active and passive knee exercises began on the 1st day postoperatively [28].

Different techniques and instruments can be used for a dome osteotomy. A curved osteotome and an angled scale guide showing the amount of correction in degrees can be used; with a transverse incision just below the TT, cortical holes are drilled with the opening facing distally and in the shape of a dome. These holes are connected to complete the osteotomy; Steinmann pins with a Charnley compression device applied in the sagittal plane fix the correction or this can also be done with an anteriorly placed external fixator.

### Focal dome osteotomy

The focal dome osteotomy is an osteotomy created with the centre of the dome coincident to the CORA (centre of rotation angulation) derived from deformity calculations. The concavity of the dome faces proximally and the osteotomy creates a large bone interface. Incisions can be performed midline longitudinal or percutaneously. The division is through a percutaneous osteotomy or with a Gigli saw. A fibular osteotomy and fixation of the tibia with an external fixator is usually done. In order to tighten the lateral collateral ligament, a distal transport of the fibula can be performed with the focal dome osteotomy. If, on the other hand, tightening of the medial collateral ligament is needed, the focal dome, open wedge, neutral wedge, open wedge-focal dome or neutral focal dome osteotomies all serve to achieve this [29]. A circular external fixator can be used for fixation in this osteotomy [30]. In the circular fixator technique, the fibular osteotomy is made at the start through a posterolateral incision 10 cm distal to the fibular head. The bone is cut in an oblique fashion in order to enable the translation in the coronal plane. Two tensioned wires and a half pin are used for the proximal ring; two tensioned wires and a half pin are applied to the distal upper ring which is positioned perpendicularly to the tibial axis. Two tensioned wires are used for the most distal ring. All wires should not engage the fibula. The tibia is osteotomized just distal to the tibial tubercle with an osteotome or a Gigli saw. The lateral and anteromedial cortex are cut with precision with the osteotome. The cutting is completed in the posterior cortex with a turning movement of the osteotome (osteoclasis). The correction is achieved under fluoroscopy with alignment of the two rings [31].

### Open wedge osteotomy

This is achieved acutely or gradually. In acute distraction, fixation devices that are available include a variety of plates: the Puddu plate, T-plate, Tomofix, wedged plate or buttress plate.

The osteotomy can be monoplanar (single plane), which usually takes the form of an oblique or transverse cut, or biplanar where the cuts are V-shaped or retrotubercle. In gradual corrections, distraction osteogenesis (callus distraction, hemicallotasis) is used with external fixators. In the description by Hernigou et al., a longitudinal incision medial to the patellar tendon is made. The sartorius, gracilis and semitendinosus tendons are cut and the tendinous portions elevated from bone. The distal superficial fibres of the medial collateral ligament are divided. The tibia is osteotomized at least 3.5 cm distal to the medial joint line, proximal to the tibial tubercle and extending laterally to the proximal fibula. The fibula or the tibio-fibular joint is left intact. A bicortical graft from the iliac crest is used to distract open the medial side but the lateral cortex is left intact. No internal or external fixation material is used when the lateral cortex is intact. The use of plate and screws to augment this technique started with cases where lateral cortex had fractured and there was postoperative displacement. A bicortical graft with 3 different base heights is used to prevent knee flexion deformity and shortening of the patellar tendon. The first and widest graft is positioned posteriorly; the middle one should be 2 mm narrower and the 3rd anterior graft should be 6 mm shorter than this first [32]. Alternatively, in order to provide support of the open wedge osteotomy, a cement block may be used and osteosynthesis achieved with a buttress plate [33]. Hernigou calculated the base height of the triangular open wedge needed for correction in different types of tibial plateaus; for every degree of correction, a recommended width of the base of the open wedge was provided [34].

**Fig. 7:**
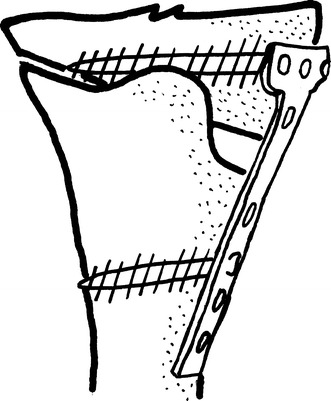
The view of the biplanar osteotomy and the fixation used by Lobenhoffer

Lobenhoffer et al. proposed rigid plate applications for stabilization in open wedge osteotomies. A diagnostic arthroscopy is performed first to evaluate for intra-articular problems. Surgery is carried out with the knee in 90° flexion. The oblique incision is made parallel to pes anserinus. The longer fibres of the medial collateral ligament are elevated subperiosteally or sharply separated from the insertion. The knee is then extended. Two K wires are inserted, parallel as viewed in an AP fluoroscopic image, starting medially from the proximal part of the insertion of pes anserinus and traversing the tibia obliquely to the level of the proximal 1/3rd of the tibio-fibular joint laterally. An oscillating saw is used to perform the osteotomy distal to the K wires, with attention to ensure division of the posteromedial cortex and preservation of the anterior cortex (continuous irrigation is also used to prevent thermal damage). Without due care, the TT can be cut anteriorly owing to the proximal location of this osteotomy. To prevent this error, the authors describe the main part of the osteotomy as occupying 70 % of the width of the tibia in a posterior-anterior direction. The cut is then deviated in the manner of a “V” in the sagittal plane so as to exit proximal to the TT and behind the patellar ligament anteriorly. The biplanar cut adds stability and avoids transection of the tibial tuberosity (Fig. 7). The depth of the osteotomy is controlled so as to leave 10 mm of bone intact on the lateral side. The osteotomy is opened through the action of a laminar spreader placed between two broad osteotomes wedged-in along the cuts. With the knee in extension, the mechanical axis is evaluated under fluoroscopic guidance with a long steel rod. The sagittal plane tibial slope is checked and the following guides used:

1. The slope is not changed if, preoperatively, there is full knee extension.
2. The slop is reduced if the extension is limited (greater opening of the wedge posteriorly).
3. The slope is reduced if there is anterior knee laxity.
4. The slope is increased if there is posterior instability or hyperextension.

Pre-contoured plates suited for the proximal part of the tibia with four locking screws for both the proximal and distal fragments are used especially for big and obese patients (TomoFix-Synthes). Bone grafting is not necessary usually. With openings exceeding 12.5–15 mm, iliac crest autograft can be used after mixing with hydroxyapatite or tricalcium phosphate [35].

Other methods of fixation have also been described. In order to fix the medial opening wedge osteotomy, Puddu developed a plate with a metal wedge that inserts into the osteotomy space. The plates have 2 and 4 holes with wedge heights ranging from 5 to 17.5 mm. A bony cut is made under the guide wires to within 1 cm medial of the lateral cortex. Wedges of different heights are driven into place in the osteotomy line. The plate with the appropriate wedge height is inserted anterior to the medial collateral ligament. Cancellous screws are used in the proximal holes whereas cortical screws for distal holes [36, 37]. In a recent revision, Puddu has produced a different plate where the posterior slope can be altered with an AP height-adjustable wedge block and incorporates a locking system of screws.

Geared plates (Osteo) have also been introduced for open wedge osteotomies. The plate consists of two pieces. It is possible to lock the plate at a desired wedge height with the aid of geared locking edges with etched measurements for guidance [38].

Koshino et al. have described a plate with a rough hydroxyapatite-coated wedge. This is inserted through an anterior incision with a retinacular release performed both laterally and medially. A transverse osteotomy 20–25 mm distal to the joint line and proximal to TT is made. Ten per cent of the lateral cortex is left intact. A 2 cm graft from the middle fibula is used as autograft. The hydroxyapatite-coated wedges of 5, 7.5 or 10 mm sizes are placed in the open wedge and two plates are used for fixation. [39].

**Fig. 8:**
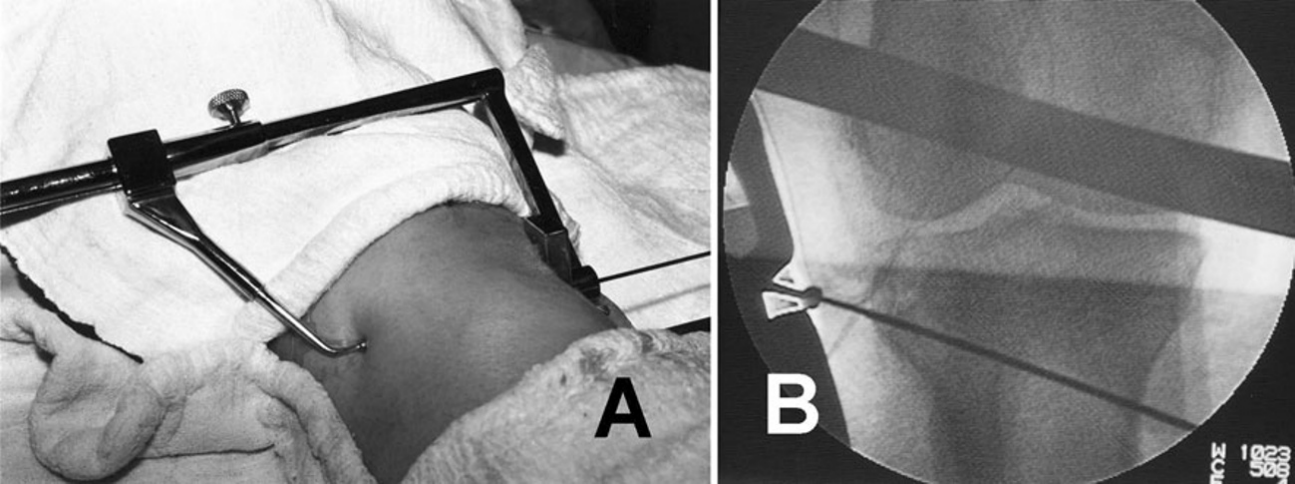
The positioning of the external guide (**a**) and fluoroscopic view of the K wire (**b**)

**Fig. 9:**
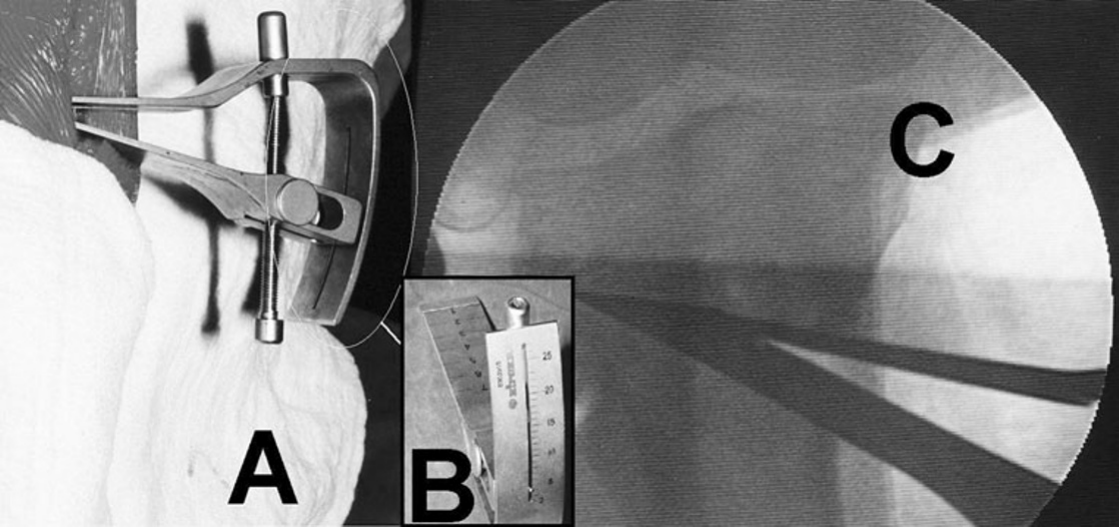
The view and the positioning of the goniometry during the surgery (**a**); the lateral view of the goniometry (**b**); the fluoroscopic view of the goniometry (**c**)

**Fig. 10:**
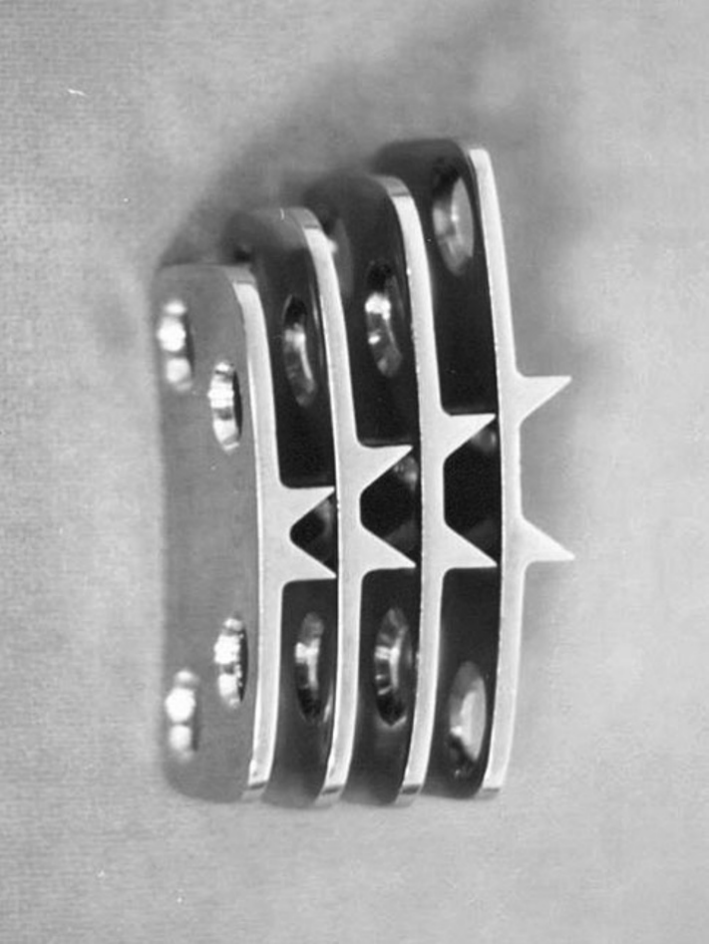
The plates used for fixation of the open wedge osteotomy

Rectangular 2- and 4-hole plates and 4-hole L-plates with wedge-shaped triangular spikes for support at the osteotomy surface have been designed by Esenkaya. These pre-contoured plates are inserted after prior diagnostic arthroscopy to allow joint debridement or chondroplasty as necessary. An anteromedial incision is used for the osteotomy and a Kirschner wire is drilled approximately 3.5–4 cm distal to the joint line with the aid of a guide (Fig. 8). The osteotomy is performed and stops 1 cm medial to the lateral cortex and 1 cm distal to the lateral tibial plateau. A distractor with a goniometer is used for controlled distraction in order to prevent fracture of the lateral tibial plateau (Fig. 9). Fixed and changeable wedges are inserted and the titanium 2- and 4-hole plates supporting the osteotomy surface are used (Fig. 10). Tricortical or bicortical autografts harvested from the ipsilateral iliac crest are used. Esenkaya has started to incorporate the modified retrotubercle osteotomy as described by Gaasbeek and Sonneveld et al. [41–47] for patients with patellofemoral disorders where the tibial tubercle is left on the proximal fragment.

### Open wedge osteotomy with callus distraction “hemicallotasis”

In contrast to acute correction, hemicallotasis is accomplished with an external fixator. Different types of external fixator can be used with this technique; a T-clamp fixator from the anterior position, a unilateral fixator from the medial side or a ring fixator can be used. Using the preserved lateral cortex as a hinge, the osteotomy is performed either under the TT. A fibular osteotomy is not necessary. The postoperative distraction starts 7–10 days at 1 mm per day until correction is achieved [40].

## Conclusion

There are several good methods of proximal tibial osteotomies for monocompartment osteoarthrosis of the knee. Good results of these techniques reflect the accrued experience of this treatment strategy. The proximal tibial osteotomy (PTO) is a joint salvage procedure. The unicompartmental knee arthroplasty is an alternative but even this has a balance of risks and benefits and is not an innocent procedure. A total knee arthroplasty is not an alternative procedure to PTO especially in young and active patients with axial malalignment secondary to medial compartment degeneration. The specific techniques and fixation devices continue to be developed. This historical evaluation serves to guide surgeons wishing to undertake this surgical correction.
